# Clinically amyopathic dermatomyositis presenting with isolated facial edema complicated by acute respiratory failure: a case report

**DOI:** 10.1186/s12891-021-03996-1

**Published:** 2021-01-28

**Authors:** Doo-Ho Lim, Min Wook So, Yeon Mee Kim, Ji Hwa Ryu, Jae Ha Lee, Chan Sun Park, Seong-Ho Kim, Sunggun Lee

**Affiliations:** 1 Department of Internal Medicine, Ulsan University Hospital, University of Ulsan College of Medicine, Ulsan, Republic of Korea; 2 Department of Internal Medicine, Pusan National University Yangsan Hospital, Gyeongnam, Republic of Korea; 3 Department of Pathology, Haeundae Paik Hospital, Inje University College of Medicine, Busan, Republic of Korea; 4 Department of Radiology, Haeundae Paik Hospital, Inje University College of Medicine, Busan, Republic of Korea; 5 Department of Internal Medicine, Haeundae Paik Hospital, Inje University College of Medicine, Haeundae-gu Haeundae-ro 875, Busan, South Korea

**Keywords:** Amyopathic dermatomyositis, Subcutaneous tissue, Edema, Pneumocystis pneumonia, Interstitial lung disease, Case report

## Abstract

**Background:**

In clinically amyopathic dermatomyositis, the hallmark cutaneous manifestations are the key to diagnosis. We report a case of clinically amyopathic dermatomyositis which presented with facial edema as the sole cutaneous manifestation and was later complicated by acute respiratory failure leading to death.

**Case presentation:**

A 58-year-old woman presented with edema of the face that had developed approximately one year ago. There was no weakness in the extremities, and the serum creatine kinase level was within normal range. On MRI, there was diffuse edematous change in the bilateral masticator and extra-ocular muscles, accompanied by subcutaneous fat infiltration in the face. A shared decision was made to defer muscle biopsy in the facial muscles. The facial swelling almost resolved with medium-dose glucocorticoid therapy but relapsed in days at glucocorticoid doses lower than 15 mg/day. Combination therapy with either azathioprine, mycophenolate, or methotrexate was not successful in maintaining clinical remission, and the swelling became more severe after relapses. A US-guided core-needle biopsy was subsequently performed in the right masseter muscle. On pathologic examination, there was a patchy CD4 + T cell-dominant lymphoplasmacytic infiltration in the stroma, necrosis of the myofibrils and prominent perifascicular atrophy. Based on those findings, a diagnosis of clinically amyopathic dermatomyositis was made. Therapy with gamma-globulin was not effective in maintaining remission. In the sixth week after starting rituximab, she presented to emergency room with altered mental state from acute respiratory failure. Despite treatment with antibiotics, glucocorticoid pulse, cyclosporin, and polymyxin B-immobilized fiber column direct hemoperfusion, she died three weeks later from persistent hypoxemic respiratory failure.

**Conclusions:**

This case showed the full spectrum and severity of internal organ involvement of dermatomyositis, although the patient presented exclusively with subcutaneous edema limited to the head. The prognosis may be more closely associated with a specific auto-antibody profile than the benign-looking initial clinical manifestation. Close follow-up of lung involvement with prophylactic treatment for Pneumocystis pneumonia and prompt implementation of emerging therapeutic regimens may improve the outcome.

## Background

Even before the classification of dermatomyositis (DM) was defined by Bohan and Peter in 1975, it was well known that there was a subgroup of patients who did not have muscle disease, regarded as amyopathic dermatomyositis (ADM)[1]. Studies have shown that there is evidence of subclinical muscle disease in MRI imaging and muscle biopsy in ADM leading to the concept of clinically amyopathic dermatomyositis (CADM), which is defined as absence of clinical muscle disease on physical examination and muscle enzyme analysis for at least six months[2]. In CADM, which represents about 11–20 % of dermatomyositis (DM)[3, 4], the hallmark cutaneous manifestations such as Heliotrope rash, Gottron’s papules, and Gottron’s sign are the key to diagnosis.

There has been a report that suggested that subcutaneous edema is a cutaneous manifestation of DM because it can be the only skin lesion at presentation[5]. In the case series, edematous DM comprised 6 % (5/86) of a DM cohort. In the muscle biopsies of the five cases of edematous DM, there were frequent microinfarctions, which were considered to be induced by vasculitis producing micro-ischemia. In the reported five cases and review of 19 reported cases, there were six cases (3/5 and 3/19, respectively) in which subcutaneous edema was the sole cutaneous manifestation, but all had clinical muscle disease, suggesting that CADM presenting exclusively with subcutaneous edema is very rare. In this study, we report a case of CADM which presented with facial edema as the sole cutaneous manifestation and was later complicated by acute respiratory failure leading to mortality.

## Case presentation

A 58-year-old woman presented with edema of the face that had developed approximately one year prior. It was not painful nor pruritic, but she felt it was more prominent on the left side. Courses of antibiotic therapy for a presumed diagnosis of cellulitis had not been effective, but glucocorticoids greatly improved the swelling, which relapsed rapidly with tapering. One week prior, as another course of glucocorticoids prescribed for a diagnosis of angioedema was tapered out, her facial edema relapsed, accompanied by tiny, pruritic vesicular lesions on the chin. Although she was evaluated in neurology and dental clinics, no specific diagnosis was made. She reported ‘dryness’ and ‘itchiness’ of the eyes, but not in the oral cavity.

**Fig. 1:**
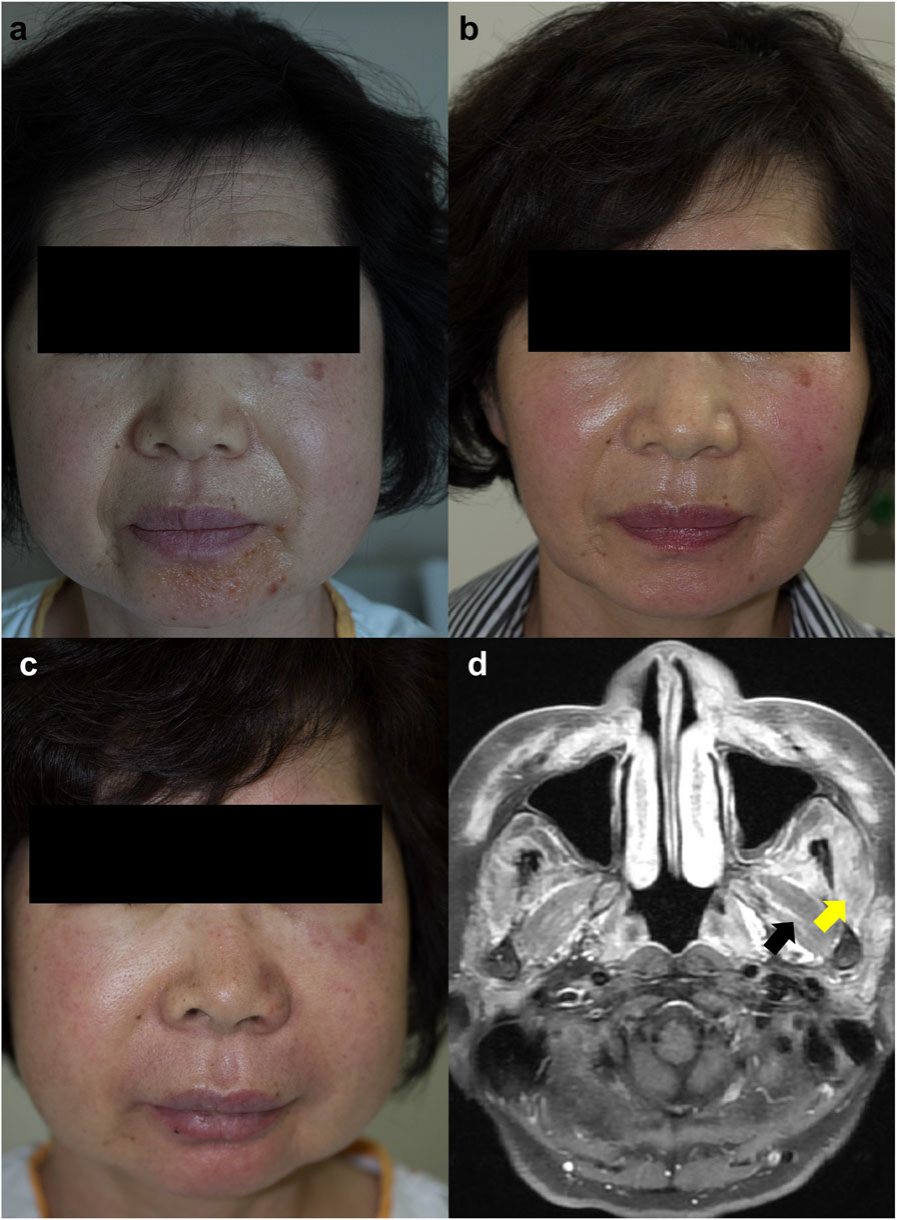
Isolated facial edema and subclinical facial muscle involvement. At initial presentation there was bilateral swelling of the face and of the left eyelid, accompanied by eczematoid eruption on the chin (**a**). After approximately two weeks of medium-dose glucocorticoid therapy the edema and the lesions were completely resolved (**b**). However, combination therapy with either azathioprine, mycophenolate, or methotrexate was not successful in maintaining remission at a prednisolone dose lower than 15 mg per day. At the last relapse, there was marked aggravation of facial and eyelid edema (**c**). Gadolinium-enhanced MRI scan of the head shows diffuse edematous change with enhancement of bilateral masticator muscles (**d**, masseter muscle [yellow arrow] and lateral pterygoid muscle [black arrow]). There was also swelling of bilateral extra-ocular muscles and edematous change with fat infiltration in the subcutaneous tissues of the anterior scalp, periorbital area, buccal space, and anterior cervical fascia (not shown)

On evaluation, there was bilateral swelling of the malar and temporal area of the face, involving the periorbital area and eyelid in the left side. On the chin, there was eczematoid vesicular eruption (Fig. 1a). There was no abnormal finding on the examination of the chest and abdomen. There was no edema or rash in the trunk or extremities. Neck flexion was preserved, and the power of the extremities’ motor function was evaluated as five on a scale of zero to five. On ophthalmologic examination, there was swelling of the left upper eyelid, and the anterior chamber was clear and deep in both eyes. Five-minute Schirmer test result was 6 mm in the right eye and 8 mm in the left eye (reference value < 5 mm for dry eye disease, but known to have low sensitivity in mild or moderate cases)[6]. Ocular staining score was 1 and 2 in the right and left eye, respectively (reference value ≥ 5 for classification of primary Sjögren’s syndrome)[7].

On the initial laboratory evaluation, there were no abnormalities, and the serum creatine phosphokinase (CPK) level was within normal range (Table 1). On serologic evaluation, antinuclear antibody was positive up to 1:160 in titer in homogeneous pattern, but anti-dsDNA Ab and anti-histone Ab was negative. Anti-SSA (> 240 U/mL) and anti-SSB (18 U/mL) antibodies were positive (fluorescent enzyme immunoassay, reference value < 7 U/mL for both); anti-RNP and anti-Smith Ab, negative. Anti-Jo1 antibody and anti-SRP antibody were negative. Anti-neutrophil cytoplasmic antibody was negative and IgG4 level was within normal range: 102 mg/dL (reference 30 ~ 201 mg/dL).

**Table 1:** Laboratory data

Variable	Reference range	At initial presentation	At the second admission
WBC	4.0 ~ 10.0 × 10^9^/L	5.2	2.84
RBC	4.2 ~ 5.4 × 10^12^/L	4.23	4.38
Hgb	12.0 ~ 16.0 g/dL	12.4	13.7
Platelet	140 ~ 440 × 10^9^/L	230	166
Differential Count			
Neutrophil	40 ~ 80 %	57.8	66.8
Lymphocyte	15 ~ 50 %	30.2	20.8
Monocyte	2 ~ 11 %	7.9	9.2
Eosinophil	0 ~ 7 %	3.5	2.8
Basophil	0 ~ 1 %	0.6	0.4
ESR	~ 120 mm	14	21
Total Protein	6.0 ~ 8.0 g/dl	7.4	7.1
Albumin	3.5 ~ 5.2 g/dl	4.3	4.1
Total Bilirubin	~ 1.2 mg/dl	0.64	1.01
AST	7 ~ 38 U/L	36	50
ALT	4 ~ 43 U/L	32	69
ALP	36 ~ 104 U/L	79	58
Glucose	70 ~ 110 mg/dl	84	109
Total Cholesterol	~ 200 mg/dl	171	204
BUN	6.0 ~ 20.0 mg/dl	16	18.6
Creatinine	0.6 ~ 1.2 mg/dl	0.79	0.82
Direct Bilirubin	~ 0.3 mg/dl	0.19	0.24
LDH	~ 250 IU/L	269	424
CPK	50 ~ 200 U/L	182	317
Uric acid	3.0 ~ 5.5 mg/dl	3.5	3.4
CRP	~ 0.30 mg/dL	0.05	0.68

*WBC* white blood cell, *RBC* red blood cell, *ESR* erythrocyte sedimentation rate, *AST* aspartate aminotransferase, *ALT* alanine aminotransferase, *ALP* alkaline phosphatase, *BUN* blood urea nitrogen, *LDH* lactase dehydrogenase, *CPK* creatine phosphokinase, *CRP* C-reactive protein

On MRI, there was a diffuse edematous change in the bilateral masticator and extra-ocular muscles, accompanied by subcutaneous fat infiltration in the buccal and periorbital spaces. There was inhomogeneous enhancement of bilateral parotid glands. On PET-CT scan there was diffuse F-18-FDG uptake in the periorbital, frontalis, temporalis, and masseter muscles and the buccal and submandibular spaces without any increased uptake in the extremities or internal organs.

The Head and Neck Surgery and Radiology departments opined that a biopsy of the muscles would entail risk of complications such as bleeding and injury to the muscles. After a discussion with the patient regarding undergoing a muscle biopsy, a presumptive clinical diagnosis of an overlap syndrome of Sjogren’s syndrome with myositis was made and treatment with a medium-dose glucocorticoid (prednisolone 30 mg/day) begun; based on reports of orbital involvement, a good response to therapy, and benign prognosis in myositis associated with Sjogren’s syndrome[8–10]. On 16th day of treatment, the facial swelling was assessed as having completely resolved (Fig. 1b); however, the facial edema relapsed during subsequent days when glucocorticoid doses were lowered to less than 15 mg/day. Combination therapy with azathioprine, mycophenolate, or methotrexate for eight months was not effective in maintaining remission at acceptable doses of glucocorticoid, and the swelling aggravated considerably with the final relapse.

The patient finally agreed to further evaluation and was admitted to the hospital. On examination, there was aggravation of the facial edema (Fig. 1c). There was swelling of the right ankle, without tenderness. Neck flexion was preserved and motor power of extremities was assessed as five on a scale of zero to five. On laboratory evaluation, there was mild elevation of liver transaminases and CPK (Table 1). Serum aldolase was 7.9 U/L (reference < 7.6 U/L). On MRI of the head, there was aggravation of the inflammation (Fig. 1d). On ultrasonography of the right ankle, there was subcutaneous edema without evidence of synovitis. A series of evaluations to screen for malignancy including tumor makers (CA19-9, CEA, and CA125), esophagogastroduodenoscopy, colonoscopy, and mammography did not reveal abnormal findings.

**Fig. 2:**
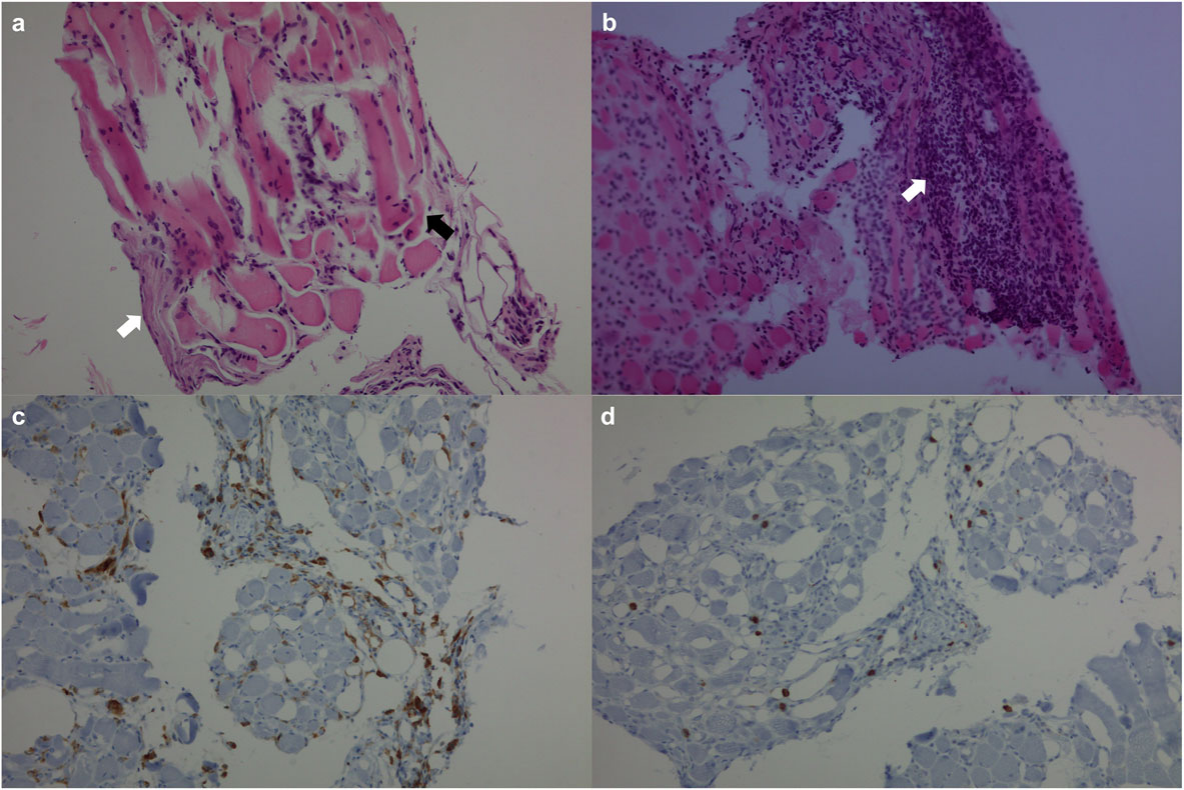
Biopsy specimens from the right masseter muscle. Hematoxylin and eosin staining shows moderate size variation of the myofibers and endomysial and perimysial fibrosis (white arrow). There is atrophy of the myofibers, mainly at the periphery of the fascicles (black arrow), which is consistent with perifascicular atrophy (**a**). Patchy lymphoplasmacytic infiltration (white arrow) is noted in the stroma (**b**), which consists of predominantly more CD4-positive cells (**c**) than CD8-positive cells (**d**) in immunohistochemical staining. All figures are at 200-fold magnification

A US-guided core-needle biopsy with an 18-gauge needle was performed in the right masseter muscle. Two pieces of tiny muscle tissue, measuring up to 0.5 × 0.1 cm, were obtained. On pathologic examination, there was moderate size variation of the myofibers, and endomysial and perimysial fibrosis. There was patchy CD4 + T cell-dominant lymphoplasmacytic infiltration in the stroma and atrophy of the myofibers, mainly at the periphery of the fascicles, being consistent with perifascicular atrophy. Necrosis of the myofibers was not prominent (Fig. 2). Based on these findings, a diagnosis of clinically amyopathic dermatomyositis was made.

**Fig. 3:**
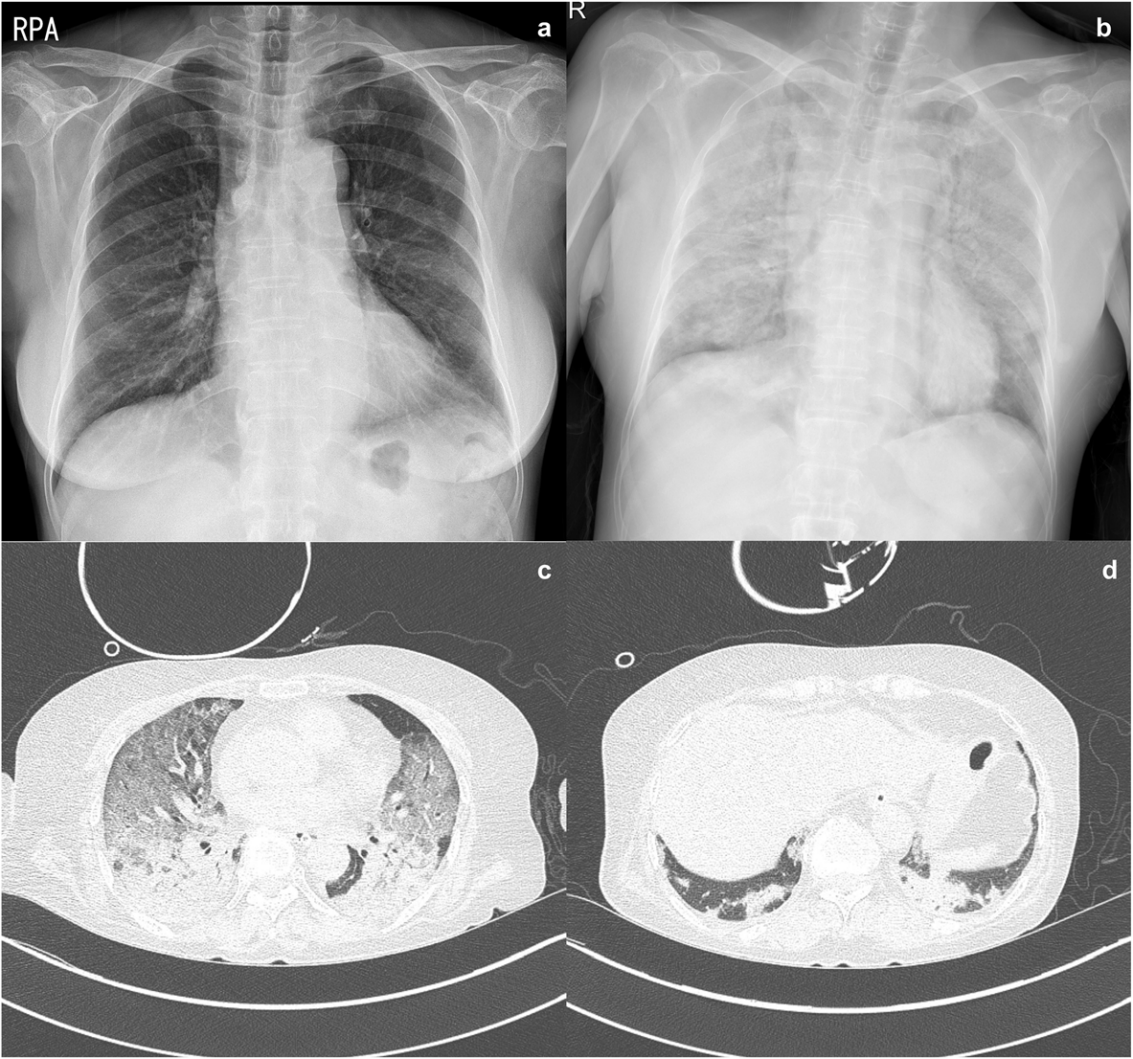
Chest radiographic images before and at the presentation of acute respiratory failure. Chest postero-anterior radiography taken about one month after initiating rituximab therapy shows no clear haziness or infiltration in both lung fields (**a**). At the presentation of acute respiratory failure with altered mentality (in the sixth week after starting rituximab) there was diffuse bilateral infiltration in both lung fields with relative sparing of both lower lung fields (**b**). Axial high-resolution computed tomography images show diffuse ground glass opacity in both upper lungs with consolidation in dependent areas (**c**); The lung base is relatively spared (**d**)

Therapy with gamma-globulin (IVIG, 2.1 g/kg per month) in combination with prednisolone 30 mg/day was initiated, and after the first cycle the facial edema had resolved completely. However, when the dose of prednisolone was decreased to 15 mg/day, she made an earlier visit to the clinic with a relapse of the facial edema within six days. By the fourth cycle of IVIG, sustained remission could not be achieved. A course of rituximab (1.0 g twice, a week apart) in combination with methylprednisolone 40 mg/day was started. In the sixth week of rituximab therapy, when glucocorticoid dose was decreased to methylprednisolone 32 mg/day, she presented to the emergency room with altered mentality that began one day prior. On presentation, there was a fever of 38.7 °C, and the patient began mechanical ventilation immediately for hypoxemic respiratory failure. On chest X-ray, there was diffuse consolidation in both lung fields (Fig. 3b). On high-resolution CT, there was diffuse consolidation in the dependent portion of the lungs and diffuse ground glass opacity with relative sparing of the base of the lungs (Fig. 3c, d). The next day, the patient underwent bronchoscopy with bronchoalveolar lavage. The total cell count was 12 × 10^6^ cells in 9 mL of fluid. Differential data of each cell type was as follows: macrophage 8 %, neutrophil 83 %, and lymphocyte 9 %. There was no bacteria isolated from the respiratory specimen, and a polymerase chain reaction (PCR) test for respiratory viruses including adenovirus, influenza virus, parainfluenza virus, and respiratory syncytial virus was negative. Pneumocystis jirovecii PCR and Gomori methenamine silver (GMS) staining were positive, and the patient began treatment with trimethoprim-sulfamethoxazole and glucocorticoids; but her hypoxemic respiratory failure progressed, and veno-venous extracorporeal (ECMO) membrane oxygenation was begun three days later. Given the lack of response to antibiotics therapy, glucocorticoid pulse and IV cyclosporine was started in consideration of rapidly progressive interstitial lung disease (RP-ILD). Over the next three weeks, despite the addition of polymyxin B-immobilized fiber column direct hemoperfusion (PMX-DHP)[11], there was no improvement in her hypoxemic respiratory failure, and according to the family’s will, the ECMO was weaned. On that day, she expired from respiratory failure.

## Discussion and Conclusions

This case, which posed significant diagnostic and therapeutic challenges, leaves important lessons for practice and research. First, isolated localized edema may be the initial manifestation of CADM. Such a case is considered very rare; we could find only one case reported in sufficient detail for us to verify the absence of muscle disease and other cutaneous manifestations, the prognosis of which was not known[12]. Second, although the patient presented initially with mild glucocorticoid-responsive facial edema, her course showed increasing extent of subcutaneous edema, decreasing response to immunomodulating therapy, and acute and severe involvement of the lungs leading to death. This suggests the prognosis cannot be predicted by the initially mild clinical manifestation alone.

Severe respiratory complications in idiopathic inflammatory myopathies such as DM pose considerable challenges to treatment, and the prognosis is poor[13, 14]. Differential diagnoses include cardiac complications, respiratory muscle weakness, drug hypersensitivity, RP-ILD, and severe and opportunistic infections[15, 16]. In many cases, it is difficult to arrive at a specific diagnosis, and there is a therapeutic dilemma of treating a severe autoimmune inflammation and a difficult-to-treat infection at the same time. Also in this case, there are a couple of explanations for the acute respiratory disease. First, the possibility of Pneumocystis pneumonia (PCP) should be considered. Prolonged glucocorticoid therapy is one of the risk factors for PCP in patients undergoing immunomodulating therapy for systemic autoimmune rheumatic diseases, and the patient was not receiving prophylaxis for it because of thrombocytopenia (< 50,000/mm^3^). Radiographic findings of acutely developed consolidation and ground glass opacity with relative sparing of the lung base is suggestive of PCP, and PCR and GMS staining were positive. In addition, even with appropriate treatment, mortality in fulminant PCP in non-HIV immunocompromised patients is high; in a recent study, the mortality rate in idiopathic inflammatory myopathy (including CADM) patients admitted to ICUs was 79.4 %, and PCP comprised approximately 20 % of the admissions[13]. Second, we believe the possibility of RP-ILD should be considered. From the early 2000s, associations between CADM and RP-ILD with poor prognosis were reported in Northeast Asia, including Korea, with mortality reaching approximately 50 %[17–19]. Later studies identified an association between anti-melanoma differentiation-associated protein 5 (MDA5) antibody and RP-ILD[20] [21], both of which are more common in CADM than in DM[22], and in Asians compared with Caucasians[21]. Recent studies report initial combination immunosuppressive therapies improved survival in RP-ILD in CADM[23], and that in the case of refractories a rescue therapy with tofacitinib may improve the outcome[24, 25]. Although we could not evaluate the presence of anti-MDA5 Ab in this case, which is a limitation of this study, rapidly progressive ILD was a possibility in the differential diagnosis of acute respiratory failure which did not respond to antibiotic therapy. Third, rituximab-induced lung disease could be considered. The patient developed acute respiratory symptoms five weeks and one day after the last rituximab infusion. This was in the period when late-onset respiratory illness may develop after rituximab infusion. However, late-onset rituximab-induced respiratory illness is less frequent than delayed-onset illness, shows a chronic course over weeks or months, and responds well to glucocorticoid therapy[26].

In conclusion, this case showed that CADM can initially manifest as a localized subcutaneous edema as the sole cutaneous manifestation but follow a progressive, refractory, and fatal course with major organ involvement, especially involving the lungs. A specific auto-antibody pattern may better predict the prognosis. Identification of the specific antibody profile and prompt initiation of therapy with proven efficacy may improve the outcome.

Authors’ contributions.

DHL analyzed clinical data and prepared the manuscript. YMK performed pathologic examination of the muscle biopsy samples and wrote the pathologic results. JHR performed the radiologic examination and muscle biopsy and contributed to writing. JHL, CSP, and SHK participated in critical care of the patient and contributed to writing. SL planned the study and performed analysis of data and literature. All authors read and approved the final manuscript.

## Data Availability

Not applicable.
